# Deregulation of inflammatory response in the diabetic condition is associated with increased ischemic brain injury

**DOI:** 10.1186/1742-2094-11-83

**Published:** 2014-05-01

**Authors:** Eunhee Kim, Aaron T Tolhurst, Sunghee Cho

**Affiliations:** 1 Burke-Cornell Medical Research Institute, White Plains, NY 10605, USA; 2 Brain & Mind Research Institute, Weill Cornell Medical College at Burke Medical Research Institute, 785 Mamaroneck Ave, White Plains, NY 10605, USA

**Keywords:** Diabetes, Inflammation, Monocytes/macrophages, Ischemic stroke, Mice

## Abstract

**Background:**

Although elicited inflammation contributes to tissue injury, a certain level of inflammation is necessary for subsequent tissue repair/remodeling. Diabetes, a chronic low-grade inflammatory state, is a predisposing risk factor for stroke. The condition is associated with delayed wound healing, presumably due to disrupted inflammatory responses. With inclusion of the diabetic condition in an experimental animal model of stroke, this study investigates whether the condition alters inflammatory response and influences stroke-induced brain injury.

**Methods:**

C57BL/6 mice were fed a diabetic diet (DD) for 8 weeks to induce an experimental diabetic condition or a normal diet (ND) for the same duration. Gene expression of inflammatory factors including monocyte chemoattractant protein-1 (MCP-1), interleukin-6 (IL-6), CCR2, and CD36 was assessed in the peripheral immune cells and brains of normal and diabetic mice before and after focal cerebral ischemia. The expression of these factors was also determined in lipopolysaccharide (LPS)-treated cultured normal and diabetic macrophages. Ischemic outcome was assessed in these mice at 3 days post-ischemia.

**Results:**

DD intervention in mice resulted in obesity and elevated insulin and glucose level in the blood. The peritoneal immune cells from the diabetic mice showed higher MCP-1 mRNA levels before and after stroke. Compared to normal mice, diabetic mice showed reduced MCP-1, IL-6, and CCR2 gene expression in the brain at 6 h post-ischemia. LPS-stimulated inflammatory responses were also reduced in the diabetic macrophages. The diabetic mice showed larger infarct size and percent swelling.

**Conclusions:**

These results showed that diabetic conditions deregulate acute inflammatory response and that the condition is associated with increased stroke-induced injury. The study suggests that interventions aimed at restoring appropriate inflammatory response in peripheral immune cells/macrophages may be beneficial in reducing stroke-induced brain injury in subjects with chronic inflammatory conditions.

## Background

In response to injury, inflammatory responses occur in a rapid and orchestrated manner, involving the innate immune system [1]. While the elicited response by mononuclear cells including monocytes/macrophages contributes to stroke-induced brain injury, an optimal level of acute inflammation by these immune cells is necessary for subsequent resolution of the inflammation and tissue repair/remodeling. Aberrant activation of these cells has been implicated in the development of age-related diseases and chronic inflammatory conditions [2,3], suggesting the importance of mounting proper acute inflammatory responses following insults.

Studies indicate that the extent of stroke-induced brain injury is influenced by the periphery. It has been shown that increased peripheral inflammation at the time of stroke aggravates ischemic injury [4]. Furthermore, exacerbated ischemic brain injury was reported in mice with elevated levels of plasma cholesterols [5]. The impact of comorbidity on stroke outcome therefore suggests the need to include comorbid conditions in the experimental animal model of stroke for a better understanding of pathology and therapeutic strategy.

Diabetes is a predisposing risk factor for cerebrovascular diseases and increases stroke incidence. The prevalent form of diabetes in the human population is type II, which accounts for approximately 90% of diabetic patients. It has been reported that 70% of stroke patients were previously diagnosed with diabetes, occult diabetes, or pre-diabetes [6]. Several clinical studies indicated that patients with diabetes had poorer ischemic outcomes [7-11].

The diabetic condition is a chronic systemic low-grade inflammation accompanied by hyperglycemia, glucose intolerance, and hyperinsulinemia [12]. Several pro-inflammatory proteins including monocyte chemoattractant protein-1 (MCP-1) and interleukin-6 (IL-6) are elevated in the plasma of diabetic patients [13,14]. As a major chemokine, MCP-1 plays a role in recruiting macrophages into adipose tissue and causing insulin resistance. The critical role of MCP-1 in the diabetic condition has been demonstrated in studies showing that its over-expression in adipocytes leads to tissue inflammation and insulin resistance, while the mouse deficient in MCP-1 or its receptor, CCR2, reverses the condition [15-17]. In addition, administration of MCP-1 in circulation elicits systemic insulin resistance [18]. Studies also indicate that diabetic conditions increase the burden of CD36 ligands via modifications of low-density lipoprotein (LDL) and excess advanced glycated end products, and augment CD36 expression. CD36 expression is increased in monocytes from type II diabetic patients and in diabetic mouse hearts, suggesting that CD36 expression is modulated by the diabetic condition [19-22].

Despite the increasing incidence of type II diabetes [12], relatively few investigations of stroke injury have been performed in animal models that closely mimic human type II diabetes. To be clinically relevant, the current study established diet-induced type II diabetes in C57BL/6 mice. Using this experimental mouse model of diabetes, this study investigates the effect of the diabetic condition on stroke-induced inflammatory response and brain injury. We report that in the diabetic condition, acute inflammatory responses are perturbed in the brain following stroke and in the macrophages after lipopolysaccharide (LPS) stimulation, and the alteration is associated with the exacerbation of stroke-induced injury.

## Methods

### Animals and diets

The use of animals and the procedures were approved by the Institutional Animal Care and Use Committee of Weill Medical College of Cornell University. Experiments were performed in male C57BL/6 mice. Six-week-old C57BL/6 mice were fed either a normal diet (ND, 4.5% fat and 53.0% carbohydrate, 5053, LabDiet, MO, USA) or a diabetogenic diet (DD, 36% fat and 35.7% carbohydrate, F3282, Bioserv, NJ, USA) for 8 weeks to induce the diabetic condition. Individuals who performed tissue cutting and gene analysis were blinded to the animal’s identity. The code was revealed after data were collected.

### Plasma glucose measurement and glucose tolerance test (GTT)

To monitor the progression of the diabetic condition, weight and fasting plasma glucose levels were measured and GTT was performed after 7 weeks of diet. Blood glucose levels were measured from a tail snip of the overnight fasted ND and DD fed mice using a glucometer (Ascensia Contour, Bayer, Germany). For GTT, the overnight fasted mice were injected intraperitoneally with 2 g/kg of D-glucose and blood glucose levels were measured at 15, 45, and 120 min post-injection.

### Plasma MCP-1 and insulin measurement

Plasma MCP-1 and insulin levels were determined using commercially available kits (MCP-1 ELISA kit, R&D systems, MN, USA; insulin EIA kit, ALPCO Diagnostics, NH, USA) according to the manufacturers’ procedures. For MCP-1, mouse trunk blood was collected in a heparinized tube. For insulin, the mice were overnight-fasted and then blood was collected from the tail vein. The collected blood was centrifuged at 3,000 rpm for 10 min and the plasma was stored at -80°C until analysis.

### Harvesting peritoneal cells and primary macrophage culture

The peritoneal cavity of normal and diabetic mice was filled with sterile phosphate buffered saline (PBS), gently massaged, and the PBS was withdrawn. The peritoneal lavage was repeated 3 to 4 times and the collected solution was centrifuged at 3,000 rpm for 10 min. The cell pellet was stored at -80°C until total RNA extraction. Primary macrophages were cultures by a modified method from previous studies [23-25]. The peritoneal cells obtained by lavage were suspended in macrophage serum-free media (MSF, Invitrogen, Carlsbad, CA, USA) containing 1% penicillin/streptomycin (Sigma, St Louis, MO, USA). The re-suspended cells were plated in a 12-well cell culture dish (2 × 10^6^ cells/well) and incubated overnight at 37°C with 5% CO_2_. The adhered cells on the plate were washed with sterile PBS, incubated in the MSF for 1 h, and then treated with 0.2 μg/mL LPS for 6 h. Total RNA was extracted from the LPS-treated cells for gene expression analysis.

### Flow cytometry analysis

Flow cytometry analysis was performed in peritoneal immune cells according to the methods previously described [26-28]. After blocking in 10% FBS for 1 h, the peritoneal cells were incubated with allophycocyanin-conjugated antibody against myeloid cells including monocyte/macrophages (CD11b, Clone M1/7) and a cocktail of phycoerythrin-conjugated antibodies (BD Biosciences, San Jose, CA, USA) against T cells (CD90.2, Clone 53–2.1), B cells (CD45R/B220, Clone RA3-6B2), NK cells (CD49b/Pan-NK cells, Clone DX5; NK1.1, Clone PK136), and granulocytes (Ly-6G, Clone 1A8) in 1% FBS. Cells were washed with PBS and passed through a 40-μm cell strainer prior to flow cytometry analysis (Accuri C6, BD Bioscience).

### Transient middle cerebral artery occlusion (MCAO)

The normal and diabetic mice were subjected to MCAO according to the method previously described [5,29]. Mice were anesthetized with isoflurane (1.5% to 2.0%) with a mixture of oxygen and nitrogen (30%/70%). A fiber optic probe was glued to the parietal bone (2 mm posterior and 5 mm lateral to the bregma) and connected to a Laser-Doppler Flowmeter (Periflux System 5010; Perimed, Järfälla, Sweden) for continuous monitoring of cerebral blood flow in the center of the ischemic territory. A 6–0 Teflon-coated black monofilament surgical suture (Doccol Co., Redland, CA, USA) was inserted into the exposed external carotid artery, advanced into the internal carotid artery, and wedged into the cerebral arterial circle to obstruct the origin of the MCA for 30 min. The filament was withdrawn to allow reperfusion. Using a rectal probe controlled by a Masterflex pump and thermistor temperature controller (Cole-Parmer, Vernon Hills, IL, USA), the animals’ body temperatures were maintained at 37 ± 0.5°C during MCAO and 1 h post-ischemia.

### Tissue section strategy for infarct volume, swelling, and gene expression measurement

To obtain tissue that contains the entire infarct territory, an unbiased stereological sampling strategy was used according to the method described in the previous study [29]. Three days after MCAO, brains were excised, frozen, and serial sections spanning about 6 mm rostrocaudal (roughly +2.8 mm and extending to -3.8 mm from bregma) were collected. The entire infarct region was cryosectioned for infarct volume measurement (20 μm thickness) and collected serially at 600 μm intervals. Infarct volume and hemispheric swelling were measured using Axiovision software (Zeiss, Germany). Infarct volume was corrected for swelling by a method described previously [30]. Tissues between the 600 μm intervals were sectioned and cut in half and collected for each hemisphere to determine mRNA levels.

### RNA extraction and gene expression analysis

Total RNA was extracted using RNeasy mini extraction kit (Qiagen, Valencia, CA, USA) for cultured peritoneal macrophages or Tri reagent (MRC, OH, USA) for brain tissues. Total RNA was reverse-transcribed using oligo (dT) primers and the SuperScript First-Strand Synthesis System (Invitrogen) according to the manufacturer’s protocol. PCR primers and probes specific for MCP-1, IL-6, CCR2, CD36, and β-actin (an internal control) were obtained as TaqMan pre-developed optimized assay reagents for gene expression (Applied Biosystems, Foster City, CA, USA). The PCR reaction was performed using TaqMan Universal PCR Mastermix, No AmpErase UNG, and 7500 Fast Real-Time PCR system (Applied Biosystems) according to the manufacturer’s protocol. Reactions were performed in 20 μL total volume and incubated at 95°C for 10 min, followed by 40 cycles of 15 sec at 95°C, and 1 min at 60°C. The results were analyzed by 7500 Fast Real-Time PCR System software (Applied Biosystems).

### Data analysis

Infarct volume and percent hemispheric swelling were reported as mean ± 95% confidence interval (CI). Gene and protein levels were reported as mean ± SEM. Gene expression levels from *in vivo* studies were presented as the β-actin normalized value according to the formula, value = 2^(Ct of β-actin-Ct of target gene)^. Gene expression levels in *in vitro* studies were reported relative to control cultures and averaged from two independent experiments. Comparison between the two groups was statistically evaluated using Student’s *t*-test. Differences were considered significant at *P* <0.05.

## Results

### Characterization of experimental mouse model of type II diabetes

**Figure 1:**
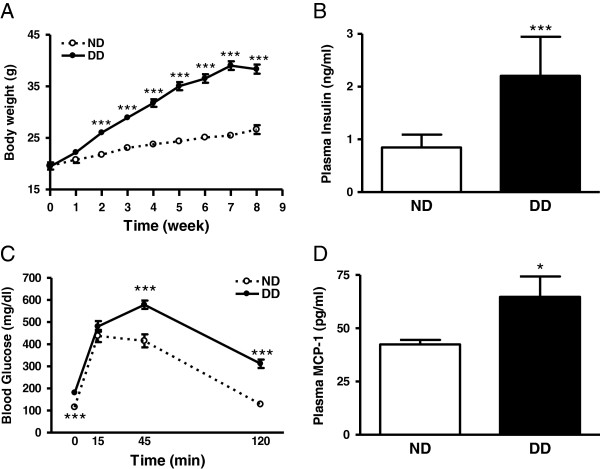
**Characterization of diet-induced diabetic mouse model. (A)** Body weight changes during 8 weeks of diet intervention, n = 14 to 15/group. **(B)** Plasma insulin levels, n = 11 to 14/group. **(C)** Clearance of blood glucose upon glucose challenge by glucose tolerance test, n = 15/group, **(D)** Plasma MCP-1 levels, n = 5/group. ND, normal diet; DD, diabetic diet; ^*^P <0.05, ^***^P <0.001 vs. ND.

Mice fed a DD gained body weight significantly faster than those fed a ND (Figure 1A). The DD also caused significantly higher plasma insulin levels measured after 7 weeks of diet (Figure 1B). DD mice displayed elevated blood glucose levels (ND vs. DD, 115.4 ± 11.5 vs. 180.7 ± 9.3, n = 15/group, *P* <0.001). Upon glucose challenge, the mice fed a DD showed slower glucose clearance, suggesting the development of insulin resistance (Figure 1C). MCP-1 levels in the plasma of DD mice were significantly higher than that of ND mice (Figure 1D). The results showed that DD intervention induces hallmarks of type II diabetes in mice.

### Elevated MCP-1 expression in diabetic peritoneal cells

**Figure 2:**
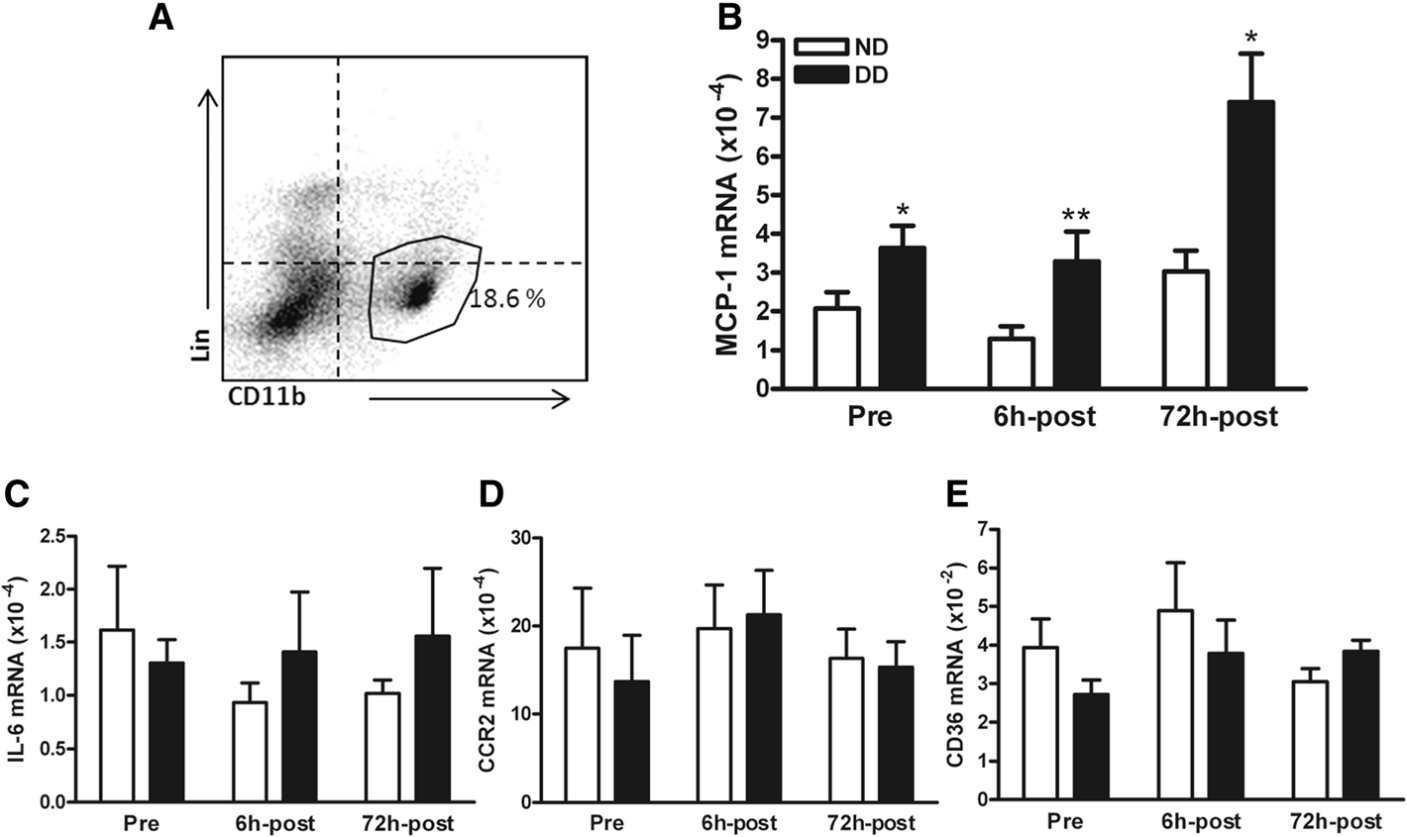
**Effect of the diabetic condition on inflammatory gene expression in the peritoneal cells before and after stroke. (A)** Flow cytometry analysis of peritoneal immune cells. The cells in the gated area indicate CD11b^+^/Lin^-^ (CD11b^high^/Lin^low^) monocytes/macrophages. **(B to E)** Gene expression of MCP-1 **(B)**, IL-6 **(C)**, CCR2 **(D)**, and CD36 **(E)** in normal and diabetic peritoneal cells prior to ischemia (pre-) and at 6 h (6 h-post) and 72 h after stroke (72 h-post), n = 9 to 13/group. ND, normal mice; DD, diabetic mice; Lin, markers for lymphocytes/NK cells/granulocytes; CD11b, a marker for mononuclear phagocytes; ^*^P <0.05, ^**^P <0.01 vs. ND.

The peritoneal cavity harbors resident immune cells including lymphocytes and macrophages. FACS analysis of the peritoneal immune cells using antibodies against CD11b (monoctyes/macrophages) and Lin, an antibody cocktail for lineage markers (lymphocytes, NK cells, and granulocytes) showed that several populations and approximately 20% of cells represent monocytes/macrophages (Lin^**low**^/CD11b^**high**^) (Figure 2A). We determined the expression of several inflammatory genes in these resident peritoneal immune cells obtained from normal and diabetic mice. Prior to stroke, the basal MCP-1 gene expression in the diabetic peritoneal cells was highly elevated (Figure 2B) while IL-1β and TNFα were not different between normal and diabetic peritoneal cells (data not shown). Additionally, we did not find differences between the groups in other inflammatory mediators including IL-6, CCR2, and CD36 (Figure 2C–E). Stroke induced an increase in MCP-1 at 6 h and 72 h after ischemia in the diabetic peritoneal cells (Figure 2B), while IL-6, CCR2, and CD36 gene expressions were not different between the normal and diabetic cells (Figure 2C–E). The results showed selective and sustained elevation of MCP-1 in the diabetic peritoneal cells.

### The compromised acute inflammatory response in the stroked brain

We next investigated stroke-induced inflammatory response in the normal and diabetic brain. Compared to that of normal mice, basal gene expression before stroke in the brain of diabetic mice showed lower CD36 (×10^-4^) (ND vs. DD, 6.9 ± 0.6 vs. 5.2 ± 0.3, n = 4 to 5/group, *P* <0.05), while MCP-1, IL-6, and CCR2 were similar between the groups (ND vs. DD, MCP-1 (×10^-5^), 6.3 ± 0.3 vs. 6.2 ± 0.5; IL-6 (×10^-5^), 4.9 ± 0.2 vs. 5.4 ± 0.4; and CCR2 (×10^-5^), 2.9 ± 0.5 vs. 3.4 ± 0.3).

**Figure 3:**
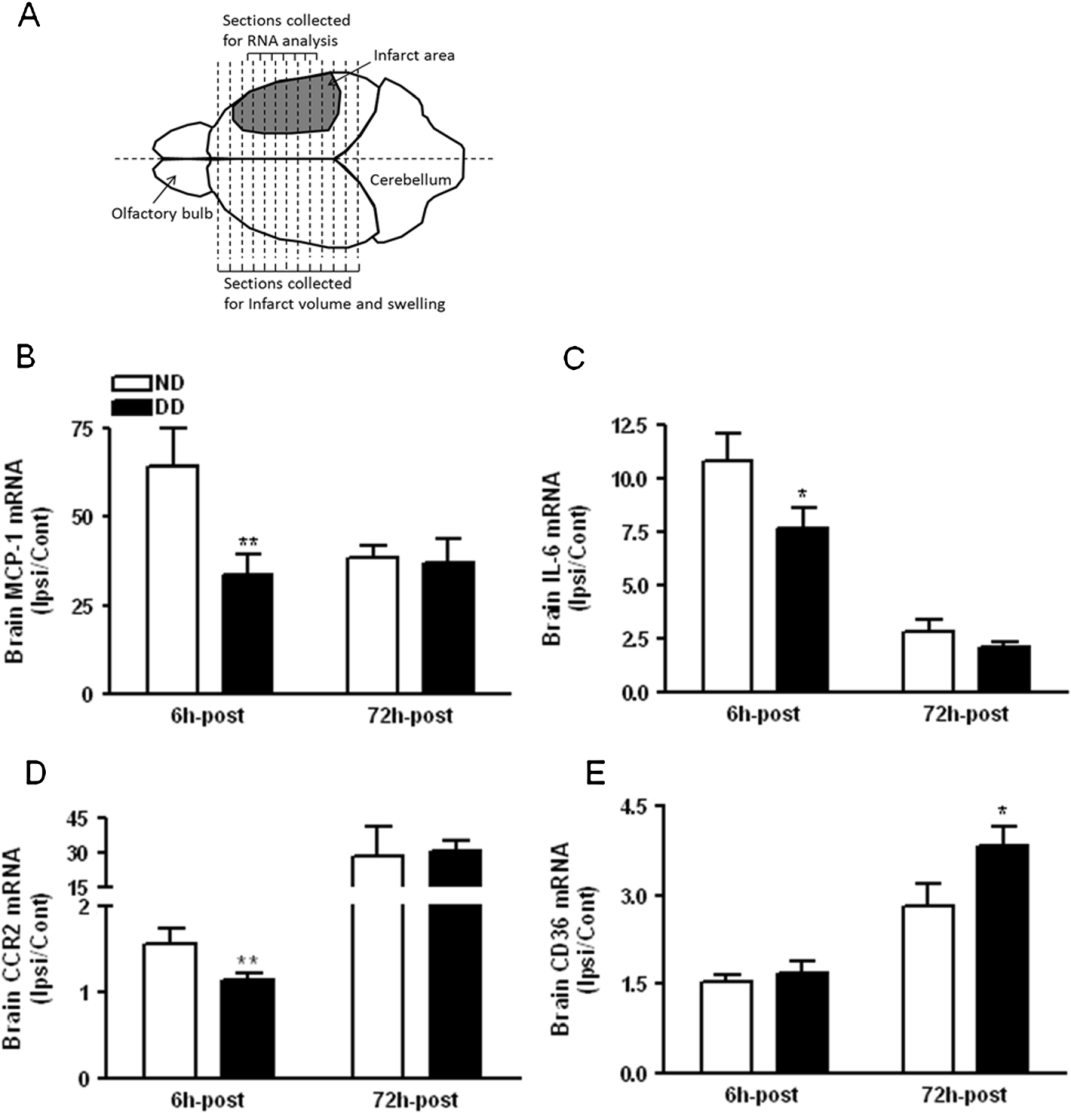
**Effect of the diabetic condition on inflammatory gene expression in the post-ischemic brain. (A)** A diagram for unbiased tissue sampling strategy. **(B to E)** Gene expressions of MCP-1 **(B)**, IL-6 **(C)**, CCR2 **(D)**, and CD36 **(E)** in the brain at 6 h (n = 9 to 12) and 72 h (n = 4 to 5) after stroke. ND, normal mice; DD, diabetic mice. Data were expressed as fold induction in the ipsilateral hemisphere relative to contralateral side (Ipsi/Cont); ^*^P <0.05, ^**^P <0.01 vs. ND.

Gene expression in the brain prior to stroke and in the contralateral hemisphere following stroke was relatively unchanged (data not shown). There was, however, a profound increase in the ipsilateral brain. MCP-1 expression increased >50-fold in the normal brains at 6 h; the fold induction at this time point was significantly attenuated in the diabetic brains (Figure 3B). This early-blunted inflammatory response at 6 h in the diabetic brain was also observed in the expression of IL-6 and CCR2, a receptor for MCP-1 (Figure 3C and D). The differences, however, were not observed at 72 h post-ischemia except in CD36, which showed increased expression in the diabetic brain (Figure 3E). Collectively, the data suggest that early inflammatory responses in the diabetic brain are deregulated.

### Attenuated LPS-stimulated inflammatory response in diabetic macrophages

**Figure 4:**
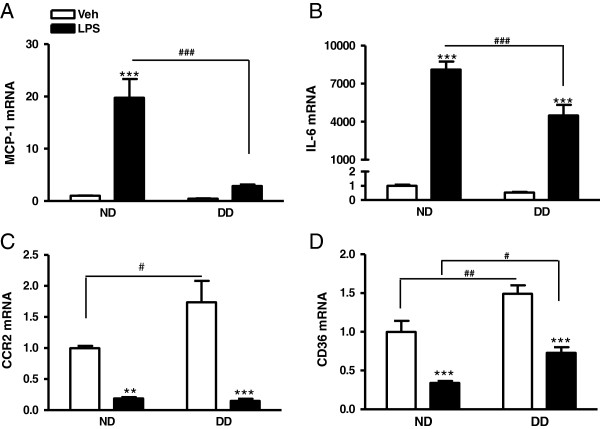
**Deregulated inflammatory response in LPS-stimulated diabetic macrophages.** Gene expression of MCP-1 **(A)**, IL-6 **(B)**, CCR2 **(C)**, and CD36 **(D)** in primary macrophages in the absence and presence of LPS. ND, macrophages from the mice fed a normal diet (n = 9); DD, macrophages from the mice fed a diabetic diet (n = 10). Data were expressed as fold difference compared to vehicle treated ND macrophages. ^**^P <0.01, ^***^P <0.001 vs. vehicle; ^#^*P* <0.05, ^##^*P* <0.01, ^###^*P* <0.001 vs. ND; two way ANOVA with a *post-hoc* Bonferroni test.

Mononuclear cells including monocytes/macrophages that infiltrate into infarct, contribute to inflammation in the injured tissue. We therefore investigated whether these immune cells largely account for the blunted inflammatory response upon insults, as we observed in the stroked brain. Primary macrophages from the normal and diabetic mice were cultured and their responses to external inflammatory stimulus were investigated. Compared to vehicle-treated cultures, LPS increased MCP-1 and IL-6 expression in normal macrophages. However, the responses to LPS were significantly attenuated in diabetic macrophages (Figure 4A and B), showing altered chemokine and cytokine expression in LPS-stimulated diabetic macrophages. CCR2 and CD36 gene expression was higher in the vehicle-treated diabetic macrophages; LPS down-regulated CCR2 and CD36 expression in both normal and diabetic macrophages (Figure 4C and D). The results suggest that diabetic macrophages exhibit altered responses upon an external inflammatory stimulus.

### Exacerbation of ischemic brain injury in the DD mice

**Figure 5:**
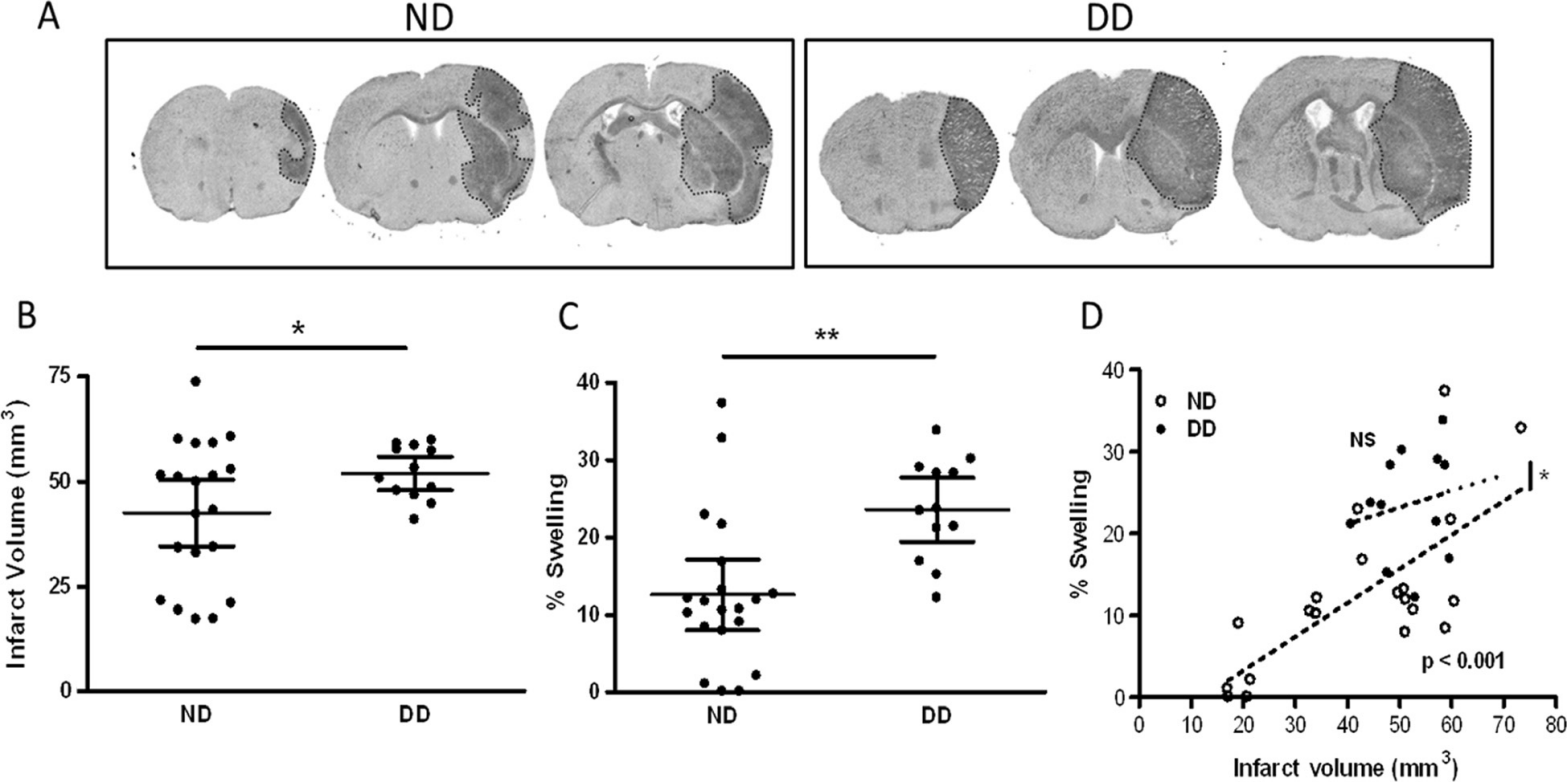
**Aggravated ischemic brain injury in the DD mice. (A)** Brain sections of normal (ND) and diabetic (DD) mice 3d-post ischemia. **(B)** Infarct volume and **(C)** percent hemispheric swelling. **(D)** Correlation between infarct volume and percent hemispheric swelling within each group. ND, n = 20; DD, n = 12; ^*^P <0.05, ^**^P <0.01 vs. ND.

The effect of diabetes on ischemic outcome was assessed. Histological examination at 3 days after stroke revealed that the diabetic mice showed an increased infarct size and percent hemispheric swelling (Figure 5A–C). To determine the contribution of hemispheric swelling, we further performed correlation analyses within the groups. Infarct size is positively correlated with percent swelling in normal mice (r^2^ = 0.5089, *P* <0.001). However, there is no correlation between infarct size and percent swelling in diabetic mice (r^2^ = 0.03895, ns). In addition, the correlation slopes between the groups were significantly different (*P* = 0.0113), showing a larger swelling component at a given infarct in the diabetic mice. The result indicates that differential dynamics in acute infarct evolution in diabetic mice may contribute to the exacerbation of stroke induced brain injury (Figure 5D).

## Discussion

Accumulating evidence suggests that peripheral status influences stroke-induced brain injury, a major CNS event. With the inclusion of the diabetic condition in an experimental animal model of stroke, the current study addresses the effect of the condition on stroke-induced inflammatory response and outcome. An important finding of this study is that the diet-induced diabetic condition captures many features of prevalent type II diabetes, evidenced by obesity, increased plasma insulin and MCP-1 protein, as well as the development of insulin resistance. In the diabetic mice, stroke-induced inflammatory response in the brain was blunted. Moreover, diabetic macrophages also displayed attenuated expression of pro-inflammatory chemokines and cytokines in response to LPS stimulation. The altered acute inflammatory response in diabetic conditions is associated with the exacerbation of stroke injury, implying the importance of mounting the proper inflammatory response in limiting stroke-induced brain injury in the presence of the comorbidity.

The diet-induced obese mice used in the current study provide a suitable experimental model to investigate the impact of the diabetes comorbidity in stroke. In contrast to the widely used genetically modified ob/ob or db/db mice that display excessively elevated fasting blood glucose (190 to 400 mg/dL) and plasma insulin levels (20-fold) [31-33], the current model displays obesity, moderately increased insulin and fasting blood glucose, insulin resistance, and increased MCP-1 in the plasma and peritoneal immune cells (Figures 1 and 2). These hallmarks of type II diabetes featured in the current model signify its validity and advantage over genetic models in studying the effect of diabetes in stroke pathology and outcome.

Hyperglycemia-induced acidosis with lactate buildup was considered a potential mechanism by which diabetic conditions exacerbate stroke-induced brain injury [34-36]. However, arguments against this view include a study that showed greater injury size in diabetic mice than in control mice despite comparable plasma glucose levels [37,38]. Moreover, a report on larger infarct size with lower glucose and lactoacidosis in male db/db mice compared to female mice [39], further supports the view that hyperglycemia, *per se*, may not account for the diabetes-induced aggravation of stroke injury.

Chronic inflammation is a salient feature of metabolic disorders and aging-related disease. Because the chronic inflammation is associated with compromised antimicrobial defenses, delayed wound healing, and impaired inflammatory responses [2,40], immunological disturbances may be an underlying event for diabetes-induced exacerbation of ischemic brain injury. Diabetic mice (*db/db*) displayed reduced inflammatory cytokine expression and microglial activation and delayed wound healing [41]. Since microglial activation and the release of chemokines and cytokines are critical steps in eliciting inflammatory response, we speculate that inability to mount a proper host immune response immediately after cerebral ischemia in diabetic microglia may cause an extended inflammatory phase, which leads to a prolonged infiltration of peripheral immune cells and worsen ischemic injury. Sustained elevation of glucose has been linked to dysregulation of normal immune function through C-type lectin-mediated immune function [42]. Our finding of attenuated stroke-induced inflammatory response in diabetic mice is also consistent with literature showing attenuated LPS-stimulated IL-6 levels and hypoxic/ischemia-induced cytokine in diabetic conditions [41,43-45]. Besides its implicated role as a prototype inflammatory receptor in acute cerebral ischemia [5,29,46], CD36 in the plasma was identified as a novel marker of insulin resistance [21,47]. Increased CD36 expression in the ischemic brains and diabetic macrophages in this study may reflect the feed-forward expression of CD36 in the presence of excess CD36 ligands such as advanced glycated end products and glucose-oxidized LDL in the diabetic condition.

Although stroke-induced MCP-1 expression suggests its role in the trafficking of inflammatory immune cells to the injury site, the attenuated MCP-1 expression in the diabetic brain following stroke (Figure 3) suggests a perturbed immune response. In age-related chronic inflammatory conditions, several functions of mononuclear phagocytes, including immune defense, inflammation, and phagocytosis, are deregulated [40]. In the current study, the reduced LPS-stimulated MCP-1 and IL-6 expression in the diabetic macrophages (Figure 4) provided a mechanistic link between impaired mononuclear phagocyte function and diabetes-induced exacerbation of ischemic injury. Despite the impaired pro-inflammatory MCP-1 and IL-6 in the ischemic brain and LPS-stimulated macrophages, increased stroke-induced brain injury in diabetic conditions suggests a benefit of rapid inflammatory response following stroke. This may be relevant to the reported protective role of MCP-1 against apoptotic stimuli and excitotoxicity in neurons [48,49], norepinephrine-induced reduction of neuronal damage [50], and wound healing [51,52].

## Conclusions

In summary, we report that disturbed immune response in diabetic mice is associated with increased stroke-induced brain injury. The study showed that impaired inflammatory function in mononuclear cells and inability to elicit rapid inflammatory responses may partly underlie the diabetes-induced exacerbation of stroke injury. As tissue injury typically elicits a rapid inflammatory response to resolve inflammation, this study indicates the importance of mounting timely inflammatory responses to limit stroke-induced injury in subjects with chronic systemic inflammatory conditions. Careful and discriminatory blockade of inflammation should be cautiously considered for stroke therapies.

## Abbreviations

DD: Diabetogenic diet; ND: Normal diet; GTT: Glucose tolerance test; IL-6: Interleukin-6; LDL: Low-density lipoprotein; LPS: Lipopolysaccharide; MCAO: Middle cerebral artery occlusion; MCP-1: Monocyte chemoattractant protein-1.

## Competing interests

We do not have competing interests.

## Author’s contributions

EK designed the experiments, established the diabetic mouse model, performed biochemical and molecular assays, performed data analysis, and wrote the manuscript. ATT generated the mouse model of ischemia and assessed stroke outcome. SC directed the overall study design, analyzed the data, and finalized the manuscript. All authors have read and approved the final manuscript.
